# Microfluidic‐ and Field‐Assisted 3D Printing: Leveraging Fluidic Control, Electrokinetic Phenomena, and Other Physical Fields to Advance Additive Manufacturing

**DOI:** 10.1002/elps.70041

**Published:** 2025-09-23

**Authors:** Guillermo Ramirez‐Alvarado, Gongchen Sun

**Affiliations:** ^1^ Department of Biomedical Engineering and Chemical Engineering University of Texas at San Antonio San Antonio Texas USA

**Keywords:** additive manufacturing | electrokinetics | microfluidics | three‐dimensional (3D) printing

## Abstract

Three‐dimensional (3D) printing has revolutionized manufacturing by enabling the rapid fabrication of complex structures, yet conventional 3D techniques remain constrained by inherent limitations in resolution, speed, and multi‐material integration. To address these challenges, emerging approaches such as microfluidic‐assisted and field‐assisted additive manufacturing have been developed to enhance the capabilities and versatility of the method. Microfluidic‐assisted 3D printing leverages controlled flow patterns for material deposition and control, material gradient formation, and advanced polymerization processes. Field‐assisted methods, including electric‐, acoustic‐, and interface‐assisted approaches, directly manipulate materials during printing to enable advanced functionalities and material properties. This review summarizes the latest advancements in microfluidic‐ and field‐assisted 3D printing, highlighting their unique advantage in overcoming current 3D printing limitations and their potential to drive innovation in applications ranging from biomedical devices to functional materials development.

## 1 Introduction

Three‐dimensional (3D) printing has revolutionized the field of manufacturing by enabling the rapid and cost‐effective fabrication of complex structures with high precision [1]. A wide range of 3D printing mechanisms have been developed, including well‐established methods such as fused deposition modeling (FDM), material jetting (MJ), stereolithography (SLA), and more specialized techniques such as two‐photon polymerization (TPP) and electrohydrodynamic printing (EHD) [2]. 3D printing technologies have resulted in innovations across several fields, from industrial applications in aerospace and automotive manufacturing to biomedical applications in prosthetics fabrication, tissue engineering, and microfluidic devices [3, 4, 5]. Unlike traditional manufacturing methods such as subtractive fabrication (e.g., silicon or glass etching) or formative techniques (e.g., soft lithography and polymer molding [6, 7]), 3D printing offers significant advantages in customization, flexibility, cost efficiency, time savings, and user accessibility [8].

Advances in micro‐ and nanoscale 3D printing have opened new opportunities in fields such as electronics, microelectromechanical systems (MEMS), and microfluidics [9, 10]. Among various 3D printing mechanisms, FDM, MJ, and SLA are the focus in high‐resolution manufacturing due to their ease of use, ability to create complex structures, and commercial availability [11]. Although all these methods share the common principle of sequential material deposition, each technique differs significantly in its capabilities. To allow critical comparison, we first briefly introduce major conventional AM techniques in this section, highlighting their advantages, while examining their inherent limitations and discussing potential strategies to overcome these challenges. In the following sections, we then focus on emerging approaches in microfluidic‐ and field‐assisted additive manufacturing (AM) that leverage fluidic control and external fields to advance manufacturing capabilities. We examine how microfluidic platforms and phenomena, through control of fluid flow and material delivery, are leveraged to expand the capabilities of AM and discuss how field‐assisted AM, by integrating external fields such as electric, acoustic, and interfacial fields into the printing process, enhances material control and helps fabricate structures beyond the capabilities of conventional methods [12, 13]. Emphasis is placed on electrokinetic‐assisted AM, where applied electric fields enable fluid and particle manipulation during fabrication. In this review, we adopt the definition of AM as described in ISO/ASTM 52900‐21, where AM is defined as a process of joining materials to make parts from 3D model data, usually layer upon layer [14]. Several additional techniques, such as those related to fiber generation and surface patterning, although they may not strictly conform to the definition, are also included as they may offer insights for future hybrid or multi‐step manufacturing.

### 1.1 Fused Deposition Modeling

FDM operates by extruding filaments, usually molten thermoplastics (materials shown in Table 1), through a heated nozzle to build structures [15]. Its affordability and ability to facilitate rapid prototyping with various filament materials make it a popular choice for rapid prototyping [16]. However, the resolution of FDM printing is often constrained by the nozzle diameter and the material flow dynamics, making it challenging to achieve fine details and smooth surface finishes [17]. For instance, commercial FDM nozzles typically range from 0.1 to 1 mm in diameter, with 0.4 mm nozzles being the most commonly used, limiting lateral resolution to approximately the nozzle diameter (∼394 ± 31 µm for most printers) [18]. The layer thickness, typically set below the nozzle orifice diameter, depends directly on the nozzle diameter and significantly influences both surface roughness and printing time, with smaller nozzle diameters generally leading to longer printing times due to reduced material deposition rates, rapid filament cooling, and localized heat accumulation [18, 19]. These inherent limitations of FDM stem from its reliance on thermomechanical extrusion of filaments through physically constrained nozzles, which fundamentally restricts both resolution and process speed. In this context, microfluidic‐assisted and field‐assisted approaches present potential pathways to overcome these challenges by decoupling material delivery from nozzle geometry and mechanical extrusion.

**TABLE 1:** Summary of key characteristics, including resolution, material compatibility, advantages, disadvantages, and representative applications, of conventional additive manufacturing techniques: Fused deposition modeling (FDM), material jetting (MJ), and stereolithography (SLA).

Print technology		Resolution	Material compatibility	Advantages	Disadvantages	Application(s)
**Conventional Methods**	**FDM**	∼100 µm range: for example, ∼0.1 × ∼0.1 mm (*XY*) × ∼0.1 mm (*Z*) [2, 6, 9, 11, 18, 19, 21, 28]	Thermoplastics (e.g., ABS, PLA, PETG, PEEK, and PMMA), composites (e.g., carbon fiber and glass reinforced filaments, polymer‐polymer, polymer‐ceramic, and polymer‐metal blends), and hydrogels [2, 3, 8, 15, 16, 19, 24, 28]	Multi‐material/color, low‐cost, rapid prototyping, simplicity, broad material options [2, 3, 11, 15, 16, 21, 28]	Low‐resolution, non‐smooth finishes, smaller nozzles limit throughput, weak mechanical properties, leaking issues, difficulty printing transparent parts [2, 3, 11, 17, 18, 19, 21, 28]	Prototyping mechanical and functional parts in diverse fields: automotive/transportation, aerospace, microfluidics, tissue engineering/biomedical, and electronics [1, 2, 3, 4, 5, 6, 9, 10, 11, 15, 18, 21, 37]
	**MJ**	<50 µm range: for example, 42 × 42 µm (*XY*) × 16 µm (*Z*) [2, 6, 9, 11, 21, 28]	UV‐curable materials (e.g., acrylic photopolymers and waxy polymers formulated from monomers and oligomers such as urethane waxes and acrylates/methacrylates, with examples including formulations for ceramics and metals (e.g., nanoparticle inks)) [2, 3, 8, 20, 24, 28]	High resolution, multi‐material/color, multi‐durometer, smooth surfaces [2, 3, 11, 21, 28, 37]	Expensive, complex droplet control, narrow proprietary photopolymers, post‐processing requirements [2, 3, 11, 20, 21, 22, 23, 24, 25, 28]	High‐resolution prototypes (e.g., transportation), multi‐material/color assemblies, microfluidics, biomedical applications, and electronics [1, 2, 3, 4, 5, 6, 9, 10, 11, 20, 21]
	**SLA**	∼30 µm range: for example, 7 × 7 µm (*XY*) × 10 µm (*Z*) [2, 6, 9, 11, 21, 26, 28, 30, 31, 32]	Photopolymers (e.g., acrylate, epoxy, and vinyl ether‐based systems made from monomers and oligomers such as polyester acrylates (PEA), epoxy acrylates (EA), urethane acrylates (UA), amino acrylates, with examples, including PEGDA) [2, 3, 8, 24, 28, 33, 35]	High‐resolution, transparent parts, broad material options, greater design freedom vs. FDM/MJ [2, 3, 11, 21, 26, 27, 28, 29, 30, 31, 34, 35]	Over‐polymerization from light attenuation, complex multi‐material integration, post‐processing requirements [2, 3, 11, 28, 32, 33, 34, 35, 36, 37]	High‐resolution prototypes (e.g., transportation), dental and medical devices, and microfluidics [1, 2, 3, 4, 5, 6, 9, 10, 11, 21, 26, 29, 31, 32, 33, 35, 36, 37]

### 1.2 Material Jetting

MJ involves the deposition of liquid photopolymer droplets that are cured layer by layer [20]. MJ excels in fabricating multi‐material, multi‐color, and multi‐durometer platforms with high resolution, with commercial systems such as the PolyJet platform having a reported resolution of 42 by 42 µm in the *XY* plane and a layer thickness down to 16 µm [21]. However, MJ systems, compared to FDM systems, are often more expensive due to the complexity of their droplet generation and control mechanisms, as well as the cost and narrow range of proprietary photopolymer resins and the need for post‐processing consumables such as support materials, solvents, and curing equipment [20, 22], summarized in Table 1. The droplet generation mechanism, deposition control, and post‐processing steps in MJ can be found in recent reviews [23, 24, 25]. In the context of this review, we highlight the fundamental constraint on the minimum achievable feature size and layer thickness due to the droplet size. The lateral resolution is further limited by drop placement accuracy and the wetting behavior of the substrate. These fundamental limitations highlight the need for alternative droplet generation methods by applying an external force (e.g., electrical) for improved control over droplet generation (smaller size/resolution) and placement (substrate wetting behavior).

### 1.3 Stereolithography

Compared with FDM and MJ, SLA offers the ability to create high‐resolution, intricate features as well as optically transparent structures [26, 27]. Like MJ, post‐processing steps, such as solvent washing, support removal, and post‐curing, are also required. In contrast, a key advantage of SLA compared to MJ is the versatility of its resin formulations. Although the mechanical properties of printed parts depend on the specific formulation, a wide range of monomers, oligomers, and photoinitiator systems have been developed to achieve resins with tailored properties such as flexibility and mechanical strength [28]. Compared to FDM, SLA offers greater design freedom, particularly in the fabrication of high‐resolution, intricate geometries, as highlighted by the development of complex microfluidic diagnostic devices that integrate multiple functions [29]. SLA typically uses a photon source, such as a laser or digital light projector, to initiate layer‐by‐layer photopolymerization of liquid photocurable resins in a vat, with each layer formed by the coordinated movement of a motorized stage [30]. By optimizing the light source and system configuration, SLA can achieve a feature resolution as fine as 15 by 15‐µm cross section in 3D enclosed microchannels, with recent advancements reporting even finer resolutions such as isoporous membranes with 7‐µm pores [26, 31]. Additional details for SLA can be found in Table 1.

Despite these advantages, SLA technologies face several challenges. First is the inherent limitations on resolution imposed by optical and photopolymerization dynamics [32]. The light penetration and attenuation beyond the printing layer can result in over‐polymerization of underlying layers, potentially blocking channels in microfluidic applications and ultimately compromising vertical void resolution in printed structures. Multi‐material printing by SLA presents another challenge due to the complexities introduced by the liquid resin environment [33]. Several SLA multi‐material printing approaches have been developed, including vat switching, dynamic fluid exchange within a single vat (resin switching), and vat‐less methods [34]. However, the risk of cross‐contamination between resins exists during material transitions, driven by surface tension that causes residual resin to adhere to vat surfaces, print platforms, or cured layers [33]. Methods to minimize cross‐contamination, such as incorporating intermittent washing steps, may affect the surface wettability and roughness of printed resins and hence result in weakened interfacial adhesion between different resin materials and interfacial air entrapment [35, 36, 37]. Physical field‐assisted and microfluidic‐assisted approaches could provide potential alternatives to address these challenges and improve multi‐material SLA printing. For instance, electrostatic fields have been widely used to modulate surface energy and control wettability through mechanisms such as electrowetting‐on‐dielectric (EWOD) and electrowetting‐on‐conductor (EWOC) [38, 39]. Similarly, acoustic or flow‐based microfluidic actuation could enable dynamic mixing or resin replacement at precise locations, helping to minimize air entrapment and enhance resin penetration into surface features, thereby promoting mechanical interlocking and adhesion.

Overall, despite the distinct strengths of conventional 3D printing methods, they remain constrained by a few overarching limitations, including non‐uniform resolution across large build areas, challenges in optimizing material properties and durability, slow fabrication speeds of layer‐by‐layer processes, and limitations in multi‐material integration within a single print [2, 40, 41, 42]. As highlighted by a recent perspective article, the relationship between 3D printing and microfluidics is bidirectional, with microfluidics playing a key role in enhancing the precision and functionality of 3D printing technologies [43]. For example, microchannel nozzles play a critical role in material dispensing for methods such as FDM and MJ. Microfluidic phenomena, such as laminar flow and droplet generation, enable spatial and temporal controlled delivery over material deposition. These principles enable enhancements in interface quality and multi‐material patterning. Microfluidic systems also offer several distinct advantages, including reduced consumption of samples and reagents and enhanced heat and mass transfer control [44]. Similarly, integration with field‐assisted control, such as electric fields, can provide complementary capabilities by enabling control over the movement and deposition of charged particles, fluids, or polymers within the printing process. For example, techniques such as EHD printing leverage electric fields to induce fluid jetting, allowing for the deposition of fine features with an exceptional resolution beyond what is typically possible with thermomechanical extrusion or droplet‐based deposition [45]. Looking ahead, the convergence of flow and field control with 3D printing can offer new opportunities to overcome inherent limitations of conventional 3D printing methods such as resolution, interfacial adhesion, and material versatility of 3D printing.

## 2 Microfluidic‐Assisted 3D Printing

Microfluidics, the science of manipulating fluids within micrometer‐scale geometries, has evolved from its origins in MEMS into a multidisciplinary field with applications spanning biology, medical science, optics, and chemical synthesis [46, 47]. The application of 3D printing into microfluidics represents a transformative shift in device fabrication. 3D printing has eliminated the need for costly photolithography facilities and allowed for the rapid prototyping of intricate 3D designs, substantially lowering costs and accelerating innovation [48]. As a result, complex microfluidic architectures, previously unattainable through traditional fabrication methods, are now accessible [9, 49]. Building on this progress, microfluidic principles themselves have also influenced fabrication techniques, exemplified by methods such as flowing lithography. Flowing lithography leverages microfluidic platforms to produce highly uniform and customizable particles [50, 51]. This approach, although not classified as 3D printing method, utilizes controlled laminar flow to create monodisperse structures with high reproducibility [52, 53, 54, 55]. By harnessing the predictable behavior of fluids within microfluidic systems, particles with consistent size, shape, and composition can be fabricated. These well‐defined structures are particularly valuable in drug delivery, where uniform particle size ensures controlled release kinetics and precise targeted delivery to specific tissues [56].

**FIGURE 1:**
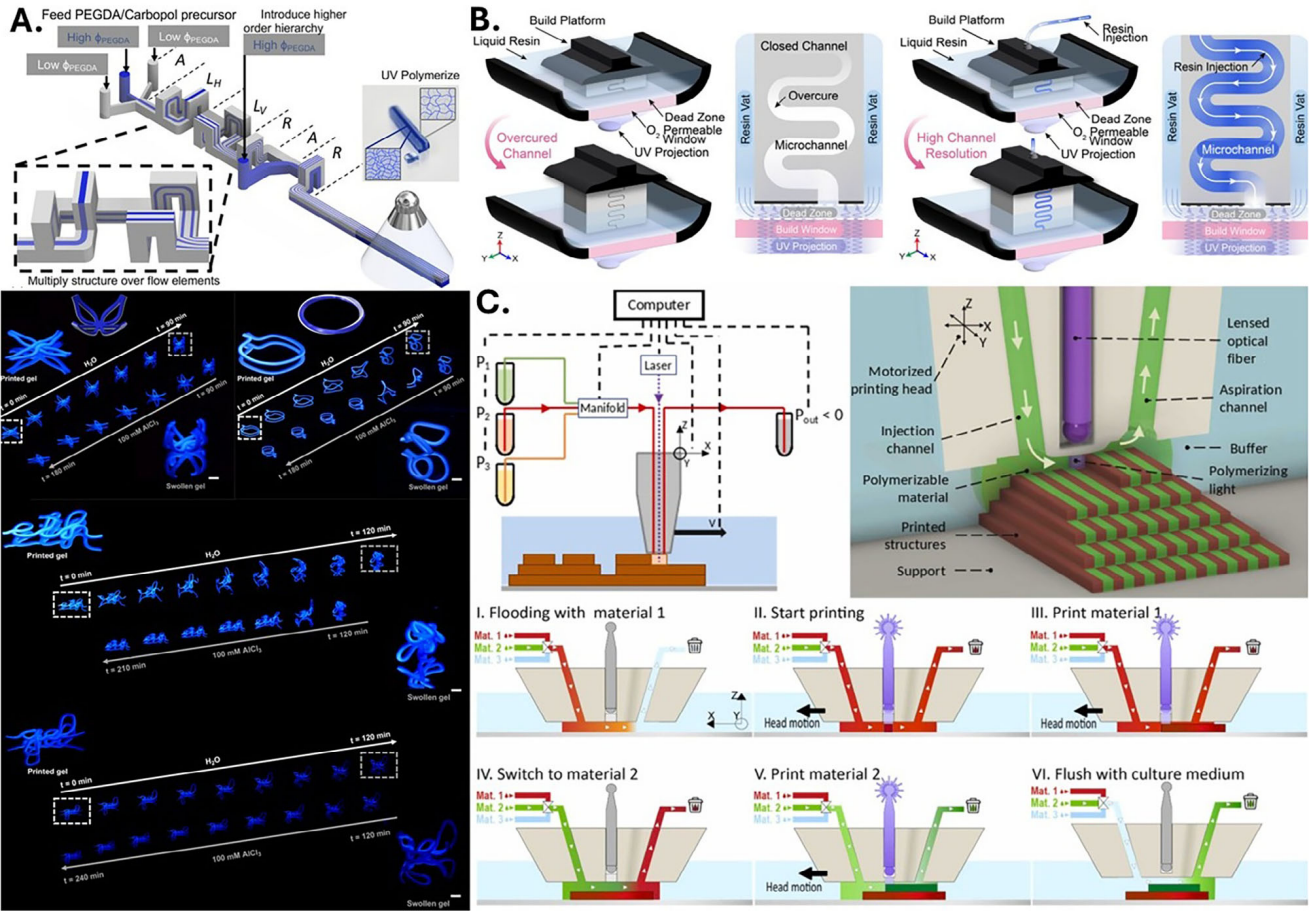
(A) Schematic of the advective assembly process, where hydrogel inks with different PEGDA volume fractions flow through a serpentine channel. The resulting hydrogel filaments exhibit comb‐like internal structures, where, upon polymerization and immersion in water, the structured hydrogels undergo controlled shape transformations. Examples include a biomimetic flower that closes, a Möbius strip that winds, and structured hydrogel lettering folding into cages. (B) Schematic of the Injection Continuous Liquid Interface Production (iCLIP) process. In conventional stereolithography, unintended UV light penetration leads to the closure of microchannels due to overcuring. In contrast, iCLIP continuously injects fresh resin through the build platform, displacing excess material and preventing overcuring, thereby enabling the fabrication of negative structures. (C) Schematic representation of the 3D‐FlowPrint concept illustrating an opto‐microfluidic printhead immersed in liquid for high‐resolution, multi‐material 3D printing. The printhead utilizes hydrodynamic flow confinement (HFC) and controlled aspiration to confine and switch materials, enabling material exchange and deposition through fluidic control. UV, Ultraviolet. *Source*: (A) Reproduced with permission from [62]; (B) reproduced from [63] under a Creative Commons Attribution 4.0 License; (C) reproduced with permission from [71].

Similarly, laminar flow principles have been applied to advance FDM technology beyond its core limitation, namely, its reliance on thermomechanical extrusion of thermoplastics through fixed‐diameter nozzles. For instance, researchers have widely integrated microfluidic‐inspired coaxial nozzles that arrange inks into core–shell morphologies [57, 58, 59, 60, 61]. These configurations enable the modification of extrusion filament compositions that were previously unprintable using conventional FDM, enabling the creation of spatially varying properties. For example, Bayles et al., although not adhering strictly to conventional 3D printing definition, introduced a novel flow‐based method for controlling the spatial distribution of cross‐linking density in polymeric hydrogels, leveraging advective assembly to achieve material structuring [62]. By channeling streams with varying macromer concentrations through modular serpentine channels (Figure 1A), they produced hydrogel filaments with tunable cross‐linking profiles. These profiles were created using horizontal and vertical layer multiplication elements, which preserved laminar flow and ensured stable interfaces between regions with differing cross‐linking densities. Upon polymerization, the resulting hydrogel filaments exhibited distinct mechanical contrasts that facilitated shape transformations, such as bending and coiling, in response to aqueous environments. A notable innovation of this method is the creation of hydrogel filaments with a “comb” cross‐linking density profile, which enabled control over differential swelling. This feature allowed for the design of actuators capable of predictable shape morphing, as demonstrated in their biomimetic flower and Möbius strip examples. This approach demonstrates how spatial control through laminar flow can create mechanically responsive architectures with heterogeneous interfacial zones. The ability to produce such complex structures could lead to potential applications in soft robotics, tissue engineering, and responsive materials. Additionally, this microfluidic advective assembly device could be integrated as a nozzle within a standard FDM printer, enabling the production of multiscale hierarchical structures in a single pass.

Another notable example of microfluidic‐assisted 3D printing is the Injection Continuous Liquid Interface Production (iCLIP) method that addresses a major challenge in SLA, the fabrication of high‐resolution negative spaces like enclosed channels [63]. As previously mentioned, overcuring, caused by ultraviolet (UV) light penetration beyond the intended polymerization layer, often leads to the closure of these spaces and limits *Z*‐axis resolution. This is especially problematic in SLA systems, where the cumulative energy dose along the *Z*‐axis often exceeds the critical threshold for polymerization, leading to the closure of voids and distortion of negative features. Coates et al. [63] pointed out that methods to mitigate overcuring exist, such as incorporating UV light‐attenuating dyes, but these introduce drawbacks like reduced transparency and the requirement for higher light intensity to solidify the resin. The iCLIP method overcomes these issues by continuously injecting fresh resin through the build platform during printing, preventing resin from overcuring (Figure 1B). This approach ensures that the cumulative UV exposure energy within negative spaces remains below the critical threshold. This flow‐based strategy disrupts SLA's direct link between light exposure and cure depth by actively replenishing resin during printing. This injection reduces the residence time of radicals and unpolymerized monomers, thereby limiting the accumulation of photonic energy and preventing the completion of cross‐linking reactions. As a result, even if transmitted light reaches into regions intended to remain void, the moving resin prevents the total absorbed energy from surpassing the critical polymerization threshold. With this approach, they successfully demonstrated 50‐µm microchannels. Applications of iCLIP have the potential to extend to the fabrication of microneedles, vascular perfusion beds for biological research, and porous media for advanced separation processes. Furthermore, this process holds potential for incorporating nonpolymerizable fluids, such as water or air, to achieve even finer negative space resolutions.

Multi‐material 3D printing methods have enabled the production of functional 3D structures integrating multiple materials. For example, centrifugal multi‐material printing utilizes an SLA‐based approach in which a rotating stage expels resins between vat‐switching steps [64]. Rotational multi‐material 3D printing (RM‐3DP) achieves layered material deposition by co‐extruding multiple viscoelastic inks through a single rotating nozzle, forming architectures such as helices or spring‐like structures [65]. In this last method, UV curing is applied on the fly to solidify components, minimizing interdiffusion between materials and showcasing a similar process to that of Bayles et al. Despite these methods, the integration of multi‐material and high‐resolution printing capabilities remains generally challenging as current conventional methods often struggle to balance the resolution needed for microscale features with the material multiplexing required for heterogeneous environments [66]. Novel approaches combining microfluidic principles with 3D printing have begun to address this challenge, usually via flowing and exchanging printing materials through the printing plane [67, 68, 69, 70]. One such innovation is the 3D‐FlowPrint concept, which integrates an opto‐microfluidic printhead immersed in a liquid [71]. The 3D‐FlowPrint system leverages microfluidic principles to control the deposition and polymerization of multiple materials through a printhead that delivers materials under hydrodynamic flow confinement (HFC) using controlled aspiration (Figure 1C). This approach enables the fabrication of sub‐millimetric to millimetric scale objects with complex geometries, including multilayered and multi‐material structures. The key component of this technology is a custom‐designed microfluidic printhead incorporating one injection and one aspiration channel, an optical fiber channel delivering 405 nm laser light directly to the printing region, a PDMS‐coated glass window at the tip that prevents adhesion, and internal microstructures such as tapered flow guides, aspiration trenches, and ridges. This open‐microfluidic architecture enables local delivery and photopolymerization of materials without immersing the entire construct in resin, avoiding vat‐switching mechanisms and reducing material use and cross‐contamination. 3D‐FlowPrint reports resolutions as fine as 10 µm in the *XY* plane and allows for rapid material switching under 60 s. The resolution of printed features is primarily determined by the ratio of laser output power to printhead velocity (P/V), which defines the local energy dose delivered to the material per unit area. This system has been successfully applied to bioprinting, where it facilitates the development of engineered microenvironments for cell culture, such as patterning cells and spheroids within 3D‐printed PEGDA hydrogel structures to create vascularized tissue models and advanced drug‐testing platforms.

Additional methods enabling 3D multi‐material printing leverage material exchange within microfluidic chambers by dynamic fluidic control. Han et al. demonstrated projection micro‐SLA (MM‐PµSL) for multi‐material printing within an enclosed chamber, utilizing fluidic control to exchange materials [72]. Mayer et al. showcased an integrated microfluidic system for laser microprinting, enabling continuous material exchange during fabrication with up to five different photoresists, demonstrating the versatility for microfluidic platforms to easily exchange material [73]. Similarly, Yoon and Park employed optofluidic maskless microfluidic lithography to fabricate microscale hydrogels within traditionally fabricated PDMS channels by sequentially exchanging resin materials and stacking layers with the aid of a press tool with a single‐axis stepper motor [74]. Their method resembles a miniaturized SLA method, where *z*‐height is mechanically controlled; however, instead of a moving build platform, a press tool is used to deform an upper PDMS before UV maskless exposure. Similarly, we have previously reported an aqueous two‐phase system (ATPS) to enable in situ 3D polymerization (IS‐3DP) within a microfluidic channel, using fluidic control to define printing layer heights and decouple *Z*‐axis control from mechanical stages [75]. Microfluidic laminar flow principle has also been explored to create distinct material layers or compositional gradients. Wang et al. utilized multi‐inlet micromixers to generate continuous gradients. By integrating a miniaturized vat and build plate platform, they fabricated 2D and 3D structures with vertical and horizontal gradients in material properties [76]. Finally, Lamont et al. demonstrated the fabrication of a five‐material DNA‐inspired microstructure by flowing different materials through a microchannel and printing with TPP [77]. This last approach, termed microfluidic multi‐material direct laser writing (µFMM‐DLW), enables sub‐micrometer resolution with integrated multi‐material patterning. Collectively, these approaches (summarized in Table 2) highlight the transformative role of microfluidics in 3D printing technologies, especially for circumventing some inherent limitations of 3D printing as well as enabling multi‐material integration.

**TABLE 2:** Summary of key characteristics, including resolution, material compatibility, advantages, disadvantages, and key capabilities of microfluidic‐assisted three‐dimensional (3D) printing methods.

	Method/Reference	Core principle	Category	Key capability
**Microfluidic‐assisted methods**	Continuous/Stop Flow Lithography [50, 51, 52, 53, 54, 55, 56, 57]	UV projection in flowing streams to produce microparticles	Lithography	High‐throughput particle fabrication
	Bayles et al. [62]	Laminar co‐flow to pattern cross‐linking gradients	Lithographic extrusion	Programmable swelling and actuation structure formation
	iCLIP [63]	Continuous resin/material injection to prevent overcuring and void closure	SLA	High‐resolution enclosed channel/void formation
	3D‐FlowPrint [71]	Hydrodynamic flow confinement with integrated optical curing	Opto‐microfluidic printhead	Multi‐material switching on print plane
	Projection micro‐stereolithography (MM‐PµSL) [72]	Projection lithography with multi‐material resin exchange through enclosed chamber	SLA with integrated chamber resin exchange	Multi‐material printing with fast material exchange
	Mayer et al. [73]	Integration of microfluidic liquid handling into two‐photon direct laser writing (DLW) to enable switching between multiple photoresists	Direct Laser Writing (TPP‐based) with resin exchange	High‐resolution (sub‐nanometer), multi‐material micropatterning
	Yoon and Park [74]	Miniaturized SLA with active resin exchange	SLA in PDMS microchannel with resin exchange	Reduced cross‐contamination and miniaturization
	In situ 3D polymerization (IS‐3DP) [75]	In situ photopolymerization of flow‐defined layers using multiphase laminar flow inside a microchannel	ATPS‐assisted lithography	Non‐mechanical *Z*‐axis control via fluid interface shaping
	Wang et al. [76]	Miniaturized SLA with active resin exchange, mixing, and gradient formation	SLA with integrated material mixing and exchange	Reduced cross‐contamination, miniaturization, and gradient formation

Abbreviations: ATPS, aqueous two‐phase system; iCLIP, Injection Continuous Liquid Interface Production; SLA, stereolithography; TPP, two‐photon polymerization.

## 3 Field‐Assisted 3D Printing

### 3.1 Electrokinetic‐Assisted Methods

Electrokinetic phenomena, rooted from interactions between fluids and charged surfaces and driven by electric fields, have become powerful tools in microfluidic technologies due to their distinct advantages as an effective, moving part‐free method for fluid and particle manipulation [78, 79]. This non‐mechanical nature provides several benefits, including enhanced robustness against mechanical failure, precise electronic control for automation, and flat velocity profiles that minimize sample dispersion. Additionally, electrokinetic systems maintain fluid and particle velocities independent of microchannel dimensions, making them highly adaptable to various applications [79]. At its core, electrokinetic motion refers to the migration of fluid or particles within a liquid solution or suspension under an applied electric field, typically generated by polarizable surfaces (electrodes) immersed in the system [80]. Commonly used electrokinetic techniques, such as electrophoresis, dielectrophoresis (DEP), and induced‐charge electrokinetics (ICEK), have been widely applied in microfluidic and lab‐on‐a‐chip devices to achieve fine fluid and particle control [81]. Unlike conventional mechanically driven printing systems, integrating electrokinetic‐assisted techniques such as DEP with AM has enabled field‐driven control on material properties and finer features [82]. Such capability allows for transition zones between materials and the creation of compositional gradients, which are essential for applications in gradient hydrogels, functional electronics, and biomimetic structures [83, 84, 85].

A well‐established example of convergence between electrokinetics and 3D printing is EHD‐based 3D printing. Similar to electrospray, which uses electric field to destabilize a liquid jet into fine charged droplets for dispersion and coating applications, EHD printing utilizes electric fields to induce ink injection from a conductive nozzle onto a substrate [86, 87]. The EHD printing process operates in two primary modes: jetting (continuous) and dripping [88], depending on the applied electric field between the nozzle and the substrate [89]. Under a sufficiently high electric potential, the liquid ink meniscus forms a stable conical shape known as a Taylor cone. Once the electrostatic force surpasses the surface tension of the liquid, material is released and deposited onto the substrate. A mobile platform or collector equipped with a three‐axis translation stage enables positioning to form intricate patterns. A closely related technique, electrospinning, also uses an electric field to form Taylor cones; however, instead of breaking into droplets, the resulting jet undergoes a sequence of bending and whipping instabilities [90, 91]. Electrospinning often employs a rotating collector to align fibers and is particularly valued for producing porous scaffolds used for tissue culture and drug delivery applications [92, 93, 94]. In contrast to the droplet‐based limitations imposed by capillary length in conventional MJ, these methods leverage electric stress to overcome droplet formation constraints, broadening material compatibility and spatial control. As such, these printing methods represent compelling electrokinetic‐assisted approaches to AM.

One of the key advantages of these printing methods is that resolution is not constrained by the nozzle's inner diameter, unlike conventional techniques such as MJ and FDM. Instead, EHD printing can produce droplets or lines significantly smaller than the nozzle diameter (sub‐micrometer), achieving high‐resolution 2D and 3D structures of up to 50 nm lateral resolution, an order of magnitude smaller than conventional MJ systems [86]. Its ability to print a wide range of inks, from conductive materials to biological solutions, makes EHD an ideal technique in the manufacturing of electronic sensors and biomedical constructs such as tissue‐engineered scaffolds [95, 96]. Furthermore, recent advancements have explored the implementation of multi‐nozzle configurations to facilitate multi‐material printing [97].

Electrowetting represents another promising electrokinetic principle for enhancing 3D printing through manipulation of surface properties. Electrowetting leverages an applied electric field to dynamically modify the wettability of a liquid on a solid surface, enabling droplet manipulation and transfer [98]. This effect is commonly implemented through EWOD configurations, in which droplets are placed atop a thin dielectric layer covering an electrode. Unlike EHD printing, electrowetting‐based printing techniques enable the formation of patterns without the need for mechanical nozzles. Recent studies have reported using electrowetting to deposit metallic materials for fabricating functional electronic circuits [99, 100].

**FIGURE 2:**
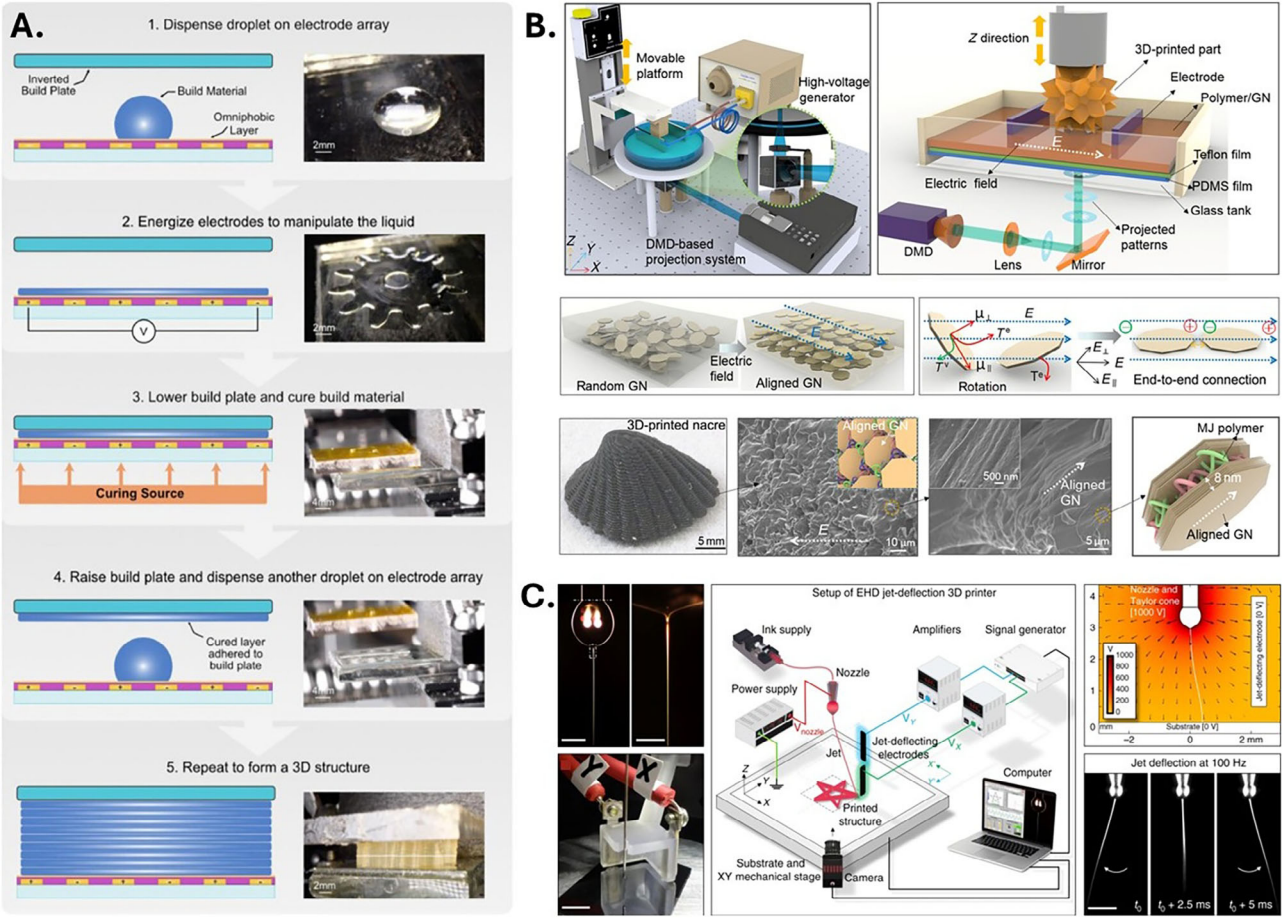
(A) Schematic representation of the Electric Field Fabrication (EFF) method, an additive manufacturing technique that employs liquid dielectrophoresis for droplet manipulation and structuring. A liquid droplet is deposited onto an electrode array, where the application of a non‐uniform electric field mobilizes and shapes it along the electrode surface. A build plate is then lowered, allowing the material to be cured and stacked layer by layer. A gear‐shaped structure is shown as an example of the 2D layer geometries achievable with this technique. (B) Schematic representation of the electrically assisted 3D printing platform for fabricating nacre‐inspired structures. Electrodes placed within the resin container generate an electric field that induces the alignment of graphene nanoplatelets (GNs) during printing. A 3D‐printed nacre‐like structure is shown, highlighting the alignment of GNs. Additionally, SEM images display the surface and cross‐sectional morphology of the printed structure. (C) Schematic representation of the electrohydrodynamic (EHD) 3D printing system with electrostatic jet deflection. The diagram includes photographs of the nozzle, ink droplet formation, Taylor cone, and the electrified jet generated by applying voltage between the nozzle and the printing substrate. Jet deflection is achieved through electrodes placed around the nozzle. The deposition process is regulated by adjusting the voltage between the nozzle, substrate, and jet‐deflecting electrodes. 3D, three‐dimensional; MJ, material jetting. *Source*: (A) Reproduced with permission from [101]; (B) reproduced from [102] under a Creative Commons Attribution License (CC BY 4.0); (C) reproduced with permission from [103].

Duncan et al. reported a novel technique called Electric Field Fabrication (EFF) that utilizes liquid DEP to manipulate and shape droplets for 3D printing [101]. This method applies a non‐uniform electric field to shape liquid droplets into desired geometries, which are subsequently cured and layered to construct the final structure (Figure 2A). The feasibility of EFF is demonstrated through the fabrication of various proof‐of‐concept components, including microfluidic channels and gears. Several advantages of the EFF platform include the printing of materials such as epoxies and resins, the potential to embed components during fabrication, the direct formation of enclosed channels without post‐processing, and reduced cross‐contamination risks. This study also acknowledges certain limitations, such as restrictions in achievable geometries due to this method favoring continuous structures, the absence of support structures, the requirement for individually patterned interdigitated electrodes for each unique slice in the build, and potential challenges related to Joule heating effects. Overall, EFF decouples printing resolution from mechanical patterning and can fabricate enclosed microfluidic geometries without the need for flushing or washing uncured resin.

Aside from spatial control of material, electrokinetic principles are also used to enhance printed material properties. Yang et al. introduced an electrically assisted 3D printing method to fabricate nacre‐inspired hierarchical structures [102]. This method integrates SLA with an applied electric field across the vat container to align graphene nanoplatelets (GNs) within a photocurable resin, forming a brick‐and‐mortar‐like structure that mimics natural nacre (Figure 2B). The aligned GNs significantly improve the mechanical properties of the printed structures, enhancing their toughness and strength compared to randomly dispersed GNs. Furthermore, the composite demonstrates anisotropic electrical conductivity, with resistance over 100 times lower along the alignment direction compared to the perpendicular direction, providing potential for real‐time structural monitoring in aerospace and biomedical applications. To demonstrate this, the authors 3D‐printed a miniaturized helmet embedded with aligned GNs connected with a light emitting diode (LED). Upon mechanical loading, gradual increases in electrical resistance were observed in response to crack formation and propagation, leading to a visibly dimmer light from the LED, providing an early warning signal prior to structural failure.

**FIGURE 3:**
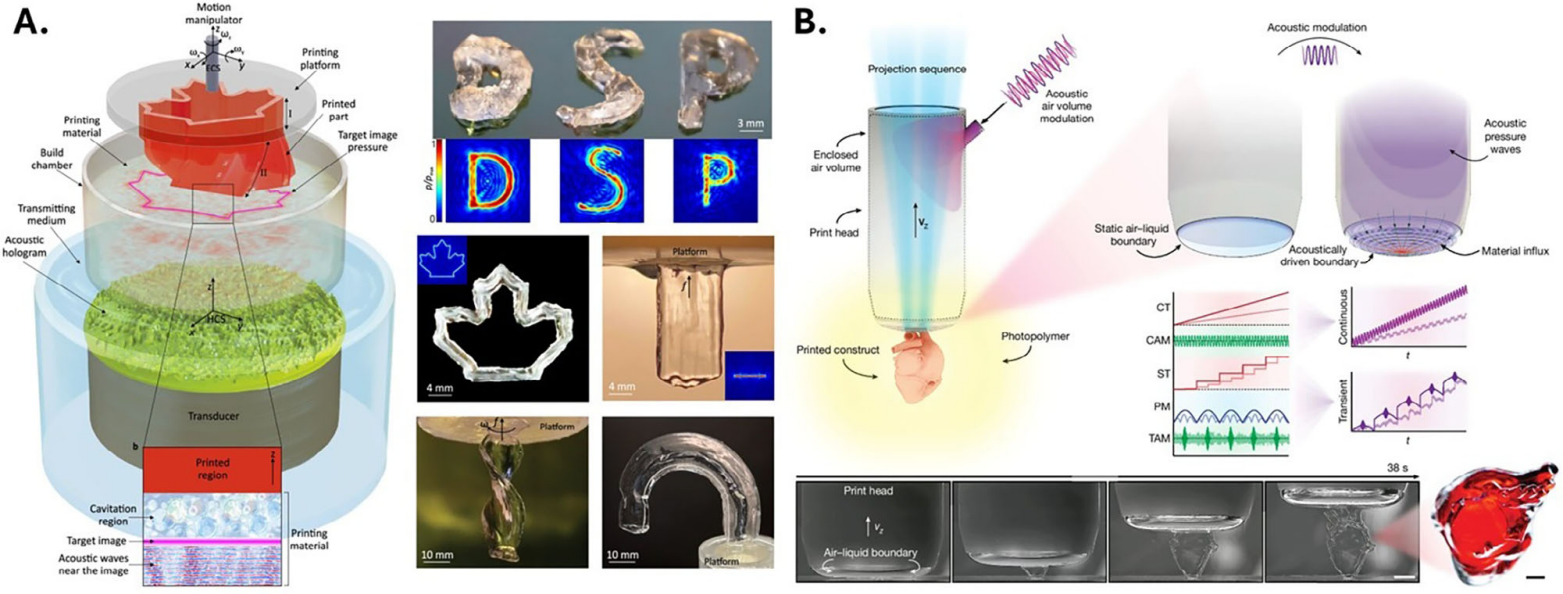
(A) Schematic representation of the Holographic Direct Sound Printing (HDSP) process. Cavitation bubbles are generated near the target pressure image to induce localized polymerization. Examples of printed structures include “DSP” letters, a maple leaf, a fully transparent extruded wall fabricated along the +*Z*‐axis, a transparent helix formed via multi‐axis motion, and a self‐supported U‐shaped object printed using a precomputed robotic trajectory. (B) Dynamic Interface Printing (DIP) setup and process. The print head, submerged in a liquid prepolymer solution, traps air and forms an air–liquid meniscus, which serves as the dynamic print interface. Visible light is projected through a glass window on the print head to selectively polymerize the prepolymer at the meniscus. The meniscus position and curvature are controlled by adjusting the internal air pressure. *Source*: (A) Reproduced from [108] under a Creative Commons Attribution‐Noncommercial‐No Derivatives 4.0 license; (B) reproduced from [110] under a Creative Commons Attribution‐NonCommercial license (CC BY‐NC).

Printing speed is another performance metric that can be enhanced by electrokinetic methods. Liashenko et al. developed an ultrafast 3D printing method with sub‐micrometer resolution by integrating electrostatic jet deflection into an EHD system [103]. This method overcomes the speed limitations of conventional nozzle‐based AM by employing high‐voltage auxiliary electrodes to electrostatically deflect the jet (Figure 3C). This method enabled 3D structure fabrication at speeds of up to 0.5 m/s in‐plane (horizontal) and 0.4 mm/s off‐plane (vertical). Compared with conventional layer‐by‐layer techniques limited by stage and curing delay, this system achieves layering rates up to 2000 layers per second, offering printing throughput orders of magnitude higher than other sub‐micron resolutions AM methods such as TPP and electron beam deposition. This technology demonstrates how electrokinetic‐assisted AM methods can integrate both high printing speeds and high resolutions, which is impossible in conventional systems such as FDM.

It has also been shown that printing resolution can be further enhanced by electrokinetic‐assisted 3D printing methods. Liu et al. introduced a metal 3D nanoprinting technique that leverages coupled electric and aerodynamic flow fields to achieve nanoscale precision [104]. The printing process uses a dual‐layer flow system, comprising an aerosol layer carrying charged nanoparticles (NPs) and a clean‐gas layer that prevents unwanted deposition. By applying a localized electric field through a nozzle, the systems enable electrophoretic focusing and deposition of selected NPs based on their electrical mobility. Additionally, this method supports multi‐material fabrication, including silver, gold, and copper. Their custom‐built nanoprinter fabricates flexible arrays of 3D metallic nanoarchitectures with feature sizes as small as 14 nm. Similarly, Jung et al. demonstrated a method that uses electrostatic focusing of charged NPs through a dielectric mask, enabling the direct fabrication of intricate 3D pure metal nanostructures without the need for polymer‐based inks [105]. Ion‐induced surface charge gradients around the mask apertures form self‐focusing electrostatic lenses, enabling the direct deposition of high‐purity metal nanostructures with feature sizes down to 85 nm.

Finally, electrokinetic‐assisted 3D printing methods have been applied in hybrid strategies. Dong et al. presented a hybrid 3D printing platform that combines thermal extrusion with electrospinning for fabricating bone scaffolds [106]. The thermal extrusion module produced scaffolds with controlled pore sizes and interconnectivity, whereas the electrospinning module deposited nanofibrous layers to mimic the extracellular matrix topography. The approach integrates a synthesized polymer, where the main scaffold structure is formed via thermal extrusion with an electrospun fiber added to enhance cell adhesion. The study demonstrated that the resulting scaffolds support cell growth, whereas the electrospun fibers improve cell adhesion and proliferation, highlighting the potential of hybrid printing strategies in tissue engineering applications. We have summarized these electrokinetic‐assisted methods in Table 3.

**TABLE 3:** Summary of key characteristics, including resolution, material compatibility, advantages, disadvantages, and key capabilities of electrokinetic‐assisted three‐dimensional (3D) printing methods.

	Method/Reference	Core principle	Category	Key capability
**Electrokinetic‐assisted methods**	EHD [84, 86, 88, 89, 95, 96, 97]	High‐voltage electric fields between a nozzle and a substrate form Taylor cones, ejecting fine jets or droplets with diameters far below the nozzle size	—	Nanometer resolution patterning with diverse selection of functional inks
	Electrospinning [85, 90, 91, 92, 93, 94]	High‐voltage electric field is applied between a nozzle and a collector to form continuous fibers	—	Fabrication of nano/microfibrous mats, aligned fibers, and scaffolds
	Electrowetting/Droplet manipulation [98, 99, 100]	Use of applied electric fields to modulate the wettability of a conductive or dielectric‐coated surface, changing the contact angle of a liquid droplet	—	Maskless, programmable droplet patterning without mechanical actuation
	Electric Field Fabrication (EFF) [101]	Non‐uniform electric field shapes liquid droplets via dielectrophoresis before curing and layering	Dielectrophoresis‐assisted at‐less printing	Vat‐less fabrication of enclosed microfluidics and embedded components
	Yang et al. [102]	SLA with applied electric field, aligning graphene nanoplatelets during curing	Electrically assisted SLA	Fabrication of strong, anisotropically conductive structures
	Liashenko et al. [103]	High‐voltage auxiliary electrodes steer and focus an EHD jet for rapid material placement; residual charge enables autofocusing	EHD printing with electrostatic jet deflection	Fast sub‐micrometer 3D printing (up to 2000 layers/s) with resolutions beyond conventional nozzle‐based AM
	Liu et al. [104]	Dual‐layer aerosol flow with localized electric fields focuses and deposits charged nanoparticles	Electric‐ and flow‐field assisted nanoparticle printing	Multi‐material 3D metallic nanoprinting with features as small as 14 nm
	Jung et al. [105]	Dielectric mask apertures generate self‐focusing electrostatic lenses for directing charged nanoparticles	Electrostatic focusing nanoparticle printing	Direct 3D printing of high‐purity metal nanostructures with features down to 85 nm

Abbreviation: EHD, electrohydrodynamic.

### 3.2 Acoustic‐Assisted Methods

Acoustic‐assisted 3D printing methods (summarized in Table 4) have been applied to modulate surface properties and induce polymerization without relying on light‐based technique, as well as to create material gradients. Surdo and Duocastella introduce an acousto‐optofluidic system that enables the generation of complex light interference patterns for maskless lithography [107]. By utilizing standing acoustic waves within a fluid‐filled cavity to modulate the refractive index, this technique creates a tunable phase grating capable of generating interference patterns in real time. This phase modulation diffracts the incident beam into multiple orders whose interference produces user‐defined intensity patterns at the image plane. The system is demonstrated with TPP, making it compatible with the photopolymers typically used in these systems, including those with radical‐based photoinitiators. The system allows on‐the‐fly pattern customization by adjusting acoustic frequencies and amplitudes without the need for mechanical adjustments to optical components. Although this surface structuring provides a way to control local surface properties, the approach's resolution depends on the optical interference fringe spacing (sub‐micron in the demonstrated setup) and the acoustic field stability. Potential uses include embedding micro‐optical textures or wettability gradients during the print, though throughput is limited by the scan/beam coverage area.

**TABLE 4:** Summary of key characteristics, including resolution, material compatibility, advantages, disadvantages, and key capabilities of acoustic‐ and interface‐assisted three‐dimensional (3D) printing methods.

	Method/Reference	Core principle	Category	Key capability
**Acoustic‐assisted methods**	Surdo and Duocastella [107]	Standing acoustic waves modulate refractive index to form tunable phase gratings, generating customizable interference patterns for TPP	Acousto‐optofluidic interference lithography	Real‐time sub‐micron surface texturing and patterning without mechanical mask changes
	Holographic Direct Sound Printing [108]	Passive acoustic holograms focus ultrasound to induce localized cavitation and instant curing across entire cross‐sections	Holographic acoustic SLA	Rapid in situ printing through opaque media or barriers with millimeter to sub‐millimeter resolution
	Acoustic Streaming‐assisted two‐photon polymerization (AS‐TPP) [109]	Acoustic streaming positions nanoparticles before final TPP curing, enabling local control of material composition	Acoustic‐field–assisted TPP	Multi‐material microstructures with micron‐scale functional gradients and anisotropic wetting properties
**Interface‐assisted methods**	Static liquid constrained interface (SLCI) SLA [110]	Uses an inert liquid boundary to minimize adhesion forces and control polymerization via oxygen inhibition	Interface‐engineered SLA	Micrometer‐resolution SLA with smooth continuous growth and support‐free overhang fabrication due to reduced mechanical adhesion forces during peeling
	Dynamic Interface Printing (DIP) [111]	Constrained air–liquid meniscus shaped by air pressure and acoustic modulation serves as the print interface	Acoustically modulated free‐surface SLA	Centimeter‐scale 3D structures within seconds without full‐volume resin exposure
	Meniscus‐enabled Projection Stereolithography (MAPS) [112]	Maintains resin meniscus via oxygen‐permeable PDMS window; resin delivered by pumps and drawn in by *z*‐stage negative pressure	Interface‐engineered vat‐free multi‐material SLA	Microscale discrete or gradient multi‐material structures with minimal resin waste

Abbreviations: SLA, stereolithography; TPP, two‐photon polymerization.

Similarly, Derayatifar et al. present Holographic Direct Sound Printing (HDSP), a sono‐chemical AM method, which projects entire cross‐sectional images of desired objects by using passive acoustic holograms inside a liquid volume [108]. The hologram patterns high‐frequency sound waves so that the pressure is focused on specific regions, triggering bubble formation and collapse. This cavitation generates high‐energy spots that cure the resin instantly (Figure 3A). Unlike earlier point Direct Sound Printing (DSP) methods that build one voxel at a time, HDSP cures an entire cross‐section in a single step, making it much faster. Walls 15 mm wide and 20 mm tall can be printed in under 90 s compared with several minutes for DSP. In terms of resolution, the smallest features are limited by the acoustic wavelength in the resin (≈370 µm at 2.28 MHz in PDMS) and by the transducer's aperture; demonstrated features were within the millimeter to sub‐millimeter feature range. The ultrasound duty cycle (DC) and acoustic power directly influence the microstructure and, consequently, the mechanical performance of HDSP‐printed parts. Infrared measurements showed that parts printed (made in PDMS) at 10% and 20% DC achieved a polymerization degree comparable to heat‐cured PDMS immediately after printing. Tensile tests indicated that the elastic modulus remained consistent with conventionally cured resin; however, the tensile strength varied depending on porosity. At low DC values (<15%), parts were dense and transparent, exhibiting higher tensile strength, whereas higher DC values produced significant cavitation‐induced porosity (>100 µm pores), leading to a reduction in tensile strength despite similar stiffness. Using higher sound frequencies improves detail but also reduces how deep the sound can penetrate due to increased attenuation. HDSP can print through opaque materials or physical barriers, enabling potential future applications within biological tissue.

Expanding the application of acoustic fields in 3D printing, Lichade et al. introduce Acoustic Streaming‐assisted TPP (AS‐TPP) to fabricate multi‐material microstructures with locally controlled material distribution [109]. This method allows selective placement of nanoparticles within printed features prior to a “locking” exposure, creating multi‐material microstructures with locally programmed wetting and mass‐transport properties. This method enables fabrication of multi‐material microstructures with micron‐scale composition control while retaining TPP's sub‐micron spatial resolution and compatibility with typical TPP resins. This method manipulates the distribution of NPs within a hydrophobic polymer matrix during printing and achieves biomimetic surfaces inspired by natural designs, such as the Namib beetle's back and rice leaves that exhibit anisotropic wetting properties. This approach is ideal for biological interfaces and lab‐on‐chip components where local chemical or functional gradients are more important than throughput.

### 3.3 Interface‐Assisted Methods

Novel 3D printing techniques leveraging interfacial phenomena have emerged as effective strategies to control and decouple the mechanical and chemical interactions between solidified structures and the printing phase (summarized in Table 4). A static liquid constrained interface (SLCI) SLA approach mitigated mechanical adhesion forces during the peeling process by employing static, inert, immiscible liquid (e.g., perfluorinated oil), so the build occurs against a liquid boundary rather than a solid plate [110]. This approach minimizes stress exerted on the printed layers, thereby improving print fidelity and, in continuous mode, accelerating the overall printing process. This interface plays two coupled roles. (i) During discrete, layer‐by‐layer operation, it behaves as a dewetting interface, so the capillary bridge of inert liquid retracts during lift, and the part experiences negligible mechanical separation forces. (ii) In continuous operation, the same liquid supplies dissolved oxygen to the resin interface, forming an oxygen‐inhibited dead zone that prevents adhesion to the window and enables smooth growth. These dual functions broaden the usable process window, with continuous mode favoring high surface quality, whereas discrete mode supports large cross‐sections and delicate overhangs. The authors demonstrated sub‐millimeter arrays of helical coils, pillars, and funnels, highlighting the ability to produce overhangs without the use of supports. In essence, SLCI leverages interfacial mechanics at the build boundary to decouple separation forces from cure kinetics and to actively tune polymerization via oxygen transport, thereby expanding design freedom while maintaining micrometer‐scale resolution and throughput.

Recently, Dynamic Interface Printing (DIP) was introduced by Vidler et al. DIP leveraged an acoustically modulated, constrained air–liquid boundary to enable the rapid generation of centimeter‐scale 3D structures within seconds [111]. In this process, a print head is submerged in a liquid prepolymer solution, creating an air–liquid meniscus that serves as the print interface, eliminating the need for full‐volume resin exposure (Figure 3B). The meniscus shape and vertical position are regulated by controlling the internal air pressure, enabling either static operation for conventional layer‐stacking or dynamic modulation at audio frequencies. DIP has been demonstrated with materials such as PEGDA and GelMA. Resolution is ultimately constrained by optical projection fidelity and meniscus stability. The reliance on free‐surface polymerization introduces sensitivity to environmental disturbances (e.g., vibrations, evaporation) that may limit suitability for nanoscale or long‐duration prints.

Similarly, Kunwar et al. introduced Meniscus‐enabled Projection Stereolithography (MAPS), a vat‐free multi‐material 3D printing platform that enables the fabrication of gradient and discrete multi‐material structures [112]. MAPS generates and maintains a resin meniscus between the cross‐linked structure and an oxygen‐permeable PDMS window. Resin is delivered via syringe pumps, optionally through an inline micromixer for gradient generation. The *z*‐stage has programmed motion that generates negative pressure to draw resin into the fabrication zone, with the pump flowing resin in between each layer. This interface‐engineered approach enables the fabrication of structures with microscale variations in mechanical stiffness, optical opacity, surface energy, magnetic NP loading, and cell density, all with minimal material waste and without the need for a resin vat.

Another novel interface‐assisted approach was reported by Lee et al., meniscus‐guided printing (MGP) for fabricating stable semi‐solid‐state polyelectrolyte‐attached liquid metal microgranular‐particle (PaLMP) structures for soft electronics [113]. Gallium‐based liquid metal materials offer exceptional electrical conductivity and mechanical deformability, making them ideal for flexible and stretchable electronic applications. However, their practical utility has been limited by challenges in achieving mechanical stability and reliable high‐resolution patterning. The MGP technique overcomes these limitations by exploiting a confined, concave liquid–air meniscus formed at the interface between the nozzle and substrate as an evaporation‐driven deposition front, enabling particle assembly and partial oxide removal in situ. As the nozzle moves, solvent evaporation is concentrated at this meniscus, causing the suspended PaLMPs to assemble densely into a continuous film, whereas acetic acid in the ink partially removes the oxide shell, electrically connecting the particles. The poly(styrene sulfonate) (PSS) coating prevents particle coalescence during deposition, improves wettability, and bridges particles for mechanical stability. This method enables the fabrication of high‐resolution patterns down to 50 µm without the need for additional post‐processing, including solvent washing and high‐temperature curing, and achieves initial conductivity and mechanical robustness in a single step. The printed structures exhibit ultra‐stretchability (up to 500% strain), robust adhesion to various substrates, and excellent electrical conductivity, making them suitable for applications such as wearable e‐skin, electronic circuits, and zero‐waste electrocardiogram (ECG) sensors.

## 4 Future Prospectives

Novel 3D printing assisted by external physical fields emerges as a growing direction in AM. Application of microscale flow phenomena and physical fields are leveraged to overcome traditional 3D printing limitations. We aimed to summarize how these novel mechanisms enhance printing by enabling spatial control of material and delivery, multi‐material integration, creation of material gradients, and modulation of surface properties that would be otherwise unattainable with conventional methods. By leveraging fluid control at the microscale, microfluidic control facilitates material displacement during printing and material switching, allowing for the integration of multiple materials within a single print. Likewise, electrokinetic methods provide sub‐micron‐level control over material deposition, making them well‐suited for applications that require nanoscale precision and selective high‐density material placement. These advancements hold the potential to bridge the gap between microscale prototyping and industrial‐scale production, enabling the fabrication of functional, high‐resolution components that are not currently manufactured through 3D printing. Field‐assisted approaches also allow tailoring material properties during printing. Acoustic‐based and interfacial manipulation techniques, such as HDSP and DIP, provide contactless methods for controlling polymerization and structure formation. By harnessing phenomena such as acoustically induced pressure fields and interface shape modulation, these methods can selectively initiate curing within 3D volumes or along dynamic liquid boundaries. This enables the fabrication of geometries and embedded features in regions that are inaccessible to conventional layer‐by‐layer or nozzle‐based approaches; for example, within sealed cavities, around pre‐existing components, or deep inside optically opaque or scattering media. Future research will likely focus on integrating multiple field‐assisted mechanisms within a single platform, achieving greater reproducibility and adaptability for various material systems. Hybrid approaches could allow the fabrication of multi‐functional structures with nanoscale features at industrial relevant scales. However, addressing scalability, developing standardized protocols for field generation, and expanding the selection of materials will further bridge the gap between laboratory demonstrations and high‐throughput manufacturing.

Additionally, integrating machine learning (ML) into field‐assisted 3D printing presents unique opportunities, including optimization of printing parameters and design generation, slicing strategies, part quality control, process efficiency, and real‐time in situ monitoring for quality assurance [114]. These microfluidic‐ and field‐assisted 3D printing often involve complex, coupled parameters that are highly sensitive to material rheology and environmental fluctuations, such as electric field strength, acoustic frequency profiles, microfluidic flow rates, and interfacial geometries. ML algorithms trained on multimodal process data (e.g., optical imaging, acoustic signals, electrical impedance, flow rate measurements) could dynamically optimize these parameters in real time, improving process stability and print fidelity. In material development, ML‐driven surrogate models could accelerate the discovery of formulations tailored to specific field‐assisted modalities, such as resins with optimal conductivity for electrokinetic deposition, reducing experimental trial‐and‐error. Predictive modeling has already shown to enhance the efficiency of multi‐material printing by identifying optimal deposition strategies for complex geometries [115]. For process control and optimization, reinforcement learning could autonomously adjust field profiles and stage trajectories to minimize cross‐contamination during material transitions or to compensate for feature distortion caused by field non‐uniformities. ML could also help overcome physical constraints such as diffraction limits or acoustic focal spot size by adaptively modifying projection patterns or field modulation schemes, hence improving resolution. Collectively, these advances point toward the development of fully autonomous and self‐correcting manufacturing platforms capable of producing highly customized, functional structures with minimal human intervention.

## Conflicts of Interest

The authors declare no conflicts of interest.

## Data Availability

Data sharing not applicable—no new data generated, or the article describes entirely theoretical research.
